# Toxic Advanced Glycation End-Products Inhibit Axonal Elongation Mediated by β-Tubulin Aggregation in Mice Optic Nerves

**DOI:** 10.3390/ijms25137409

**Published:** 2024-07-05

**Authors:** Hayahide Ooi, Ayako Furukawa, Masayoshi Takeuchi, Yoshiki Koriyama

**Affiliations:** 1 Graduate School and Faculty of Pharmaceutical Sciences, Suzuka University of Medical Science, 3500-3 Minamitamagaki, Suzuka 513-8670, Japan; ghyhd1246@gmail.com (H.O.); furukawa@suzuka-u.ac.jp (A.F.); 2 Department of Advanced Medicine, Medical Research Institute, Kanazawa Medical University, 1-1 Daigaku, Uchinada-Machi, Kahoku 920-0293, Japan; takeuchi@kanazawa-med.ac.jp

**Keywords:** toxic advanced glycation end-products, glyceraldehyde, axonal elongation, β-tubulin, tau

## Abstract

Advanced glycation end-products (AGEs) form through non-enzymatic glycation of various proteins. Optic nerve degeneration is a frequent complication of diabetes, and retinal AGE accumulation is strongly linked to the development of diabetic retinopathy. Type 2 diabetes mellitus is a major risk factor for Alzheimer’s disease (AD), with patients often exhibiting optic axon degeneration in the nerve fiber layer. Notably, a gap exists in our understanding of how AGEs contribute to neuronal degeneration in the optic nerve within the context of both diabetes and AD. Our previous work demonstrated that glyceraldehyde (GA)-derived toxic advanced glycation end-products (TAGE) disrupt neurite outgrowth through TAGE–β-tubulin aggregation and tau phosphorylation in neural cultures. In this study, we further illustrated GA-induced suppression of optic nerve axonal elongation via abnormal β-tubulin aggregation in mouse retinas. Elucidating this optic nerve degeneration mechanism holds promise for bridging the knowledge gap regarding vision loss associated with diabetes mellitus and AD.

## 1. Introduction

Axonal damage represents an early-stage neurodegenerative disorder within the central nervous system (CNS). The retina and optic nerve, being accessible regions of the CNS, offer unique substrates for investigating the impact of retinal ganglion cell (RGC) injury on optic nerve axons. The injury to RGCs, coupled with the antero-/retro-grade loss of RGC axons, manifests as a characteristic pathological alteration. Hence, comprehending the molecular mechanisms underlying axonal degeneration holds significant scientific importance. Advanced glycation end-products (AGEs) form under hyperglycemic conditions via a non-enzymatic Maillard reaction between proteins and/or amino acids and reducing sugars [1]. The formation and subsequent accumulation of AGEs in various tissues progress during normal aging, with a notably accelerated pace in type 2 diabetes mellitus (T2DM). Considering that the incidence of Alzheimer’s disease (AD) surpasses 2–5 times in patients with T2DM [2], numerous studies have explored whether T2DM serves as a clinical risk factor for the onset and progression of AD [3,4]. Several investigations have delved into the mechanisms underlying AGE-induced neurotoxicity to elucidate the pathological mechanisms of T2DM-related neurodegeneration. In diabetic retinopathy, AGEs impede normal cellular functions such as axonal transport and intracellular protein trafficking [5,6]. Conversely, optic neuropathy, encompassing optic nerve degeneration and RGC loss, has been documented in AD retinas [7,8]. Optic neuropathy in AD correlates with AGE-dependent cell death [9]. Nonetheless, no reports of AGE-dependent axonal degeneration are associated with AD or diabetes mellitus (DM).

In a previous study, we demonstrated that glyceraldehyde (GA), a metabolic intermediate of glucose (Glu) and fructose, elicits AD-like changes, including axonal degeneration, and elevates the levels of total and phosphorylated tau protein in an AGE-dependent manner [10]. We reported that GA-derived AGEs exhibit robust neurotoxicity [11] and that GA-AGEs display greater neurotoxic effects compared to Glu-AGEs in neuronal cultures, thus designating them as toxic AGEs (TAGE) [1]. TAGE have been detected in axons and intracellular neuronal cells within the hippocampus and parahippocampal gyrus in patients with AD [12]. Recently, through proteomics analysis, we identified β-tubulin as one of the proteins targeted by TAGE [13]. Microtubules consist of repeating units of heterodimers between α-tubulin and β-tubulin, and their assembly is a crucial event implicated in axon outgrowth in in vitro models such as SH-SY5Y, a human neuroblastoma cell line. As tau phosphorylation diminishes its binding to microtubules, GA-induced tau phosphorylation leads to axonal degeneration via TAGE-mediated abnormal aggregation of β-tubulin in SH-SY5Y cells [13]. However, the impact of TAGE–β-tubulin on axonal elongation in the adult mouse optic nerve, particularly in a model utilizing intraocular injection, remains unexplored. Hence, we utilized zymosan, known to induce axonal regeneration in adults following optic nerve injury [14,15], to investigate the effects of GA on zymosan-induced axonal elongation in adult mice after optic nerve injury. The findings of this study hold promise for developing novel therapeutic strategies against TAGE-related axonal degeneration disorders within the CNS.

## 2. Results

### 2.1. Detection of TAGE Proteins and Identification of TAGE–β-Tubulin in the Retina after GA Treatment

**Figure 1:**
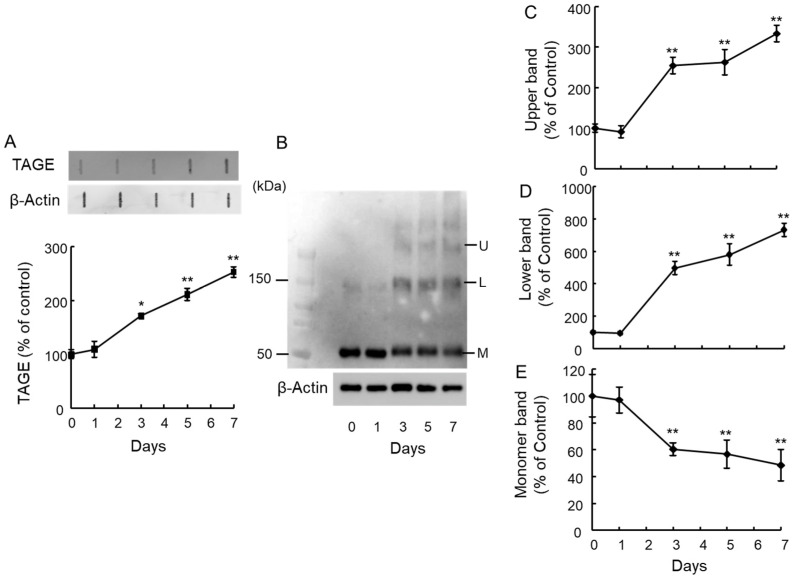
GA increased TAGE–β-tubulin and β-tubulin aggregation in the retina in a time-dependent manner. (**A**) TAGE levels were measured using slot blot analysis with an anti-TAGE antibody. The graph shows the intensity of the TAGE band in the slot blot. ** *p* < 0.01, * *p* < 0.05 vs. day 0 (n = 3). (**B**–**E**) Level of β-tubulin aggregation detected using an anti-β-tubulin antibody. (**B**) Western blot obtained using the anti-β-tubulin antibody: U: Upper band, L: Lower band, M: Monomer band. (**C**) The intensity of the upper β-tubulin bands upon GA treatment. (**D**) The intensity of the lower β-tubulin bands upon GA treatment. (**E**) The intensity of the monomer β-tubulin bands upon GA treatment. ** *p* < 0.05 vs. day 0 (n = 3).

In a previous study, we demonstrated that 1 mM GA induced TAGE formation in SH-SY5Y cells [13]. To investigate this further, we examined if intraocular injection of 1 mM GA could trigger TAGE formation. GA treatment significantly increased TAGE levels starting 3 days post-injection (Figure 1A). Interestingly, we observed a significant decrease in the levels of the 55 kDa monomeric β-tubulin protein from day 3 onwards following GA treatment (Figure 1B,E). Conversely, the intensity of aggregated β-tubulin bands increased in a time-dependent manner (Figure 1B,D: Lower band, Figure 1C: Upper band).

### 2.2. Colocalization of TAGE and β-Tubulin in the Retina after GA Treatment

**Figure 2:**
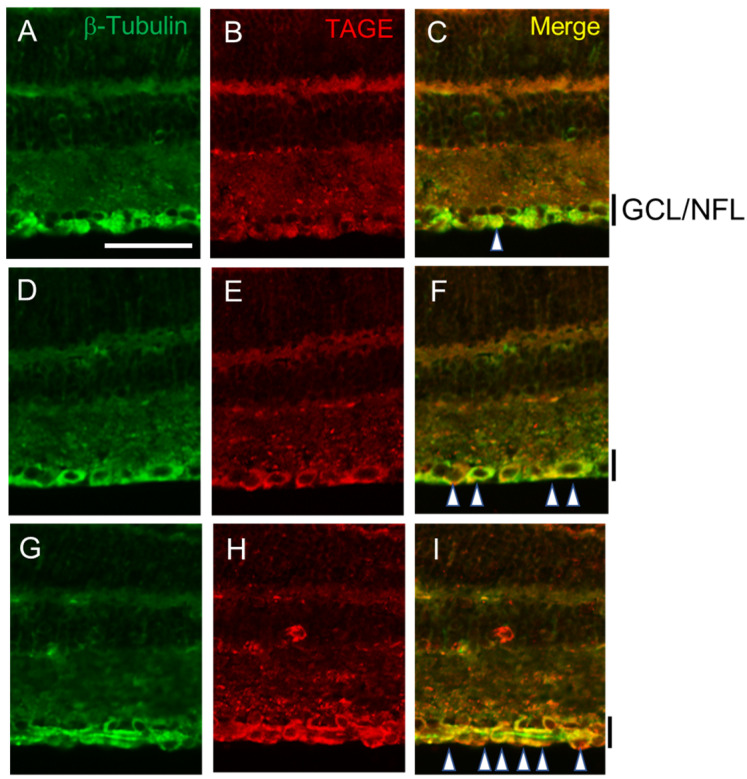
TAGE colocalized with β-tubulin in the retina upon intraocular injection of GA. (**A**,**D**,**G**) Immunoreactivity of β-tubulin was increased in GCL and NFL at 1–3 days after intraocular injection of GA (**A**) day 0, (**D**) day 1, (**G**) day 3. (**B**,**E**,**H**) Immunoreactivity of TAGE, (**B**) day 0, (**E**) day 1, (**H**) day 3. (**C**,**F**,**I**) Merged images. Arrowhead: Colocalization of TAGE and β-tubulin. GCL: ganglion cell layer, NFL: nerve fiber layer. Scale = 100 μm.

We employed immunohistochemistry to investigate the colocalization of TAGE and β-tubulin within the retina (Figure 2). Positive β-tubulin staining, primarily localized in the ganglion cell layer (GCL) and nerve fiber layer (NFL), was observed at day 0 (Figure 2A). Notably, neither the levels nor the localization of β-tubulin appeared to be significantly altered following intraocular GA treatment at 1 day (Figure 2D) or 3 days (Figure 2G). Consistent with β-tubulin distribution, weak TAGE signals were observed in the GCL and NFL of untreated retinas on day 0 (Figure 2A–C). However, strong TAGE immunoreactivity was evident from day 1 (Figure 2E) to day 3 (Figure 2H) following GA treatment, compared to that on day 0 (Figure 2B). Notably, the increased TAGE signal (Figure 2B,E,H) in the GCL colocalized with the β-tubulin layer (Figure 2A,D,G). Merged images further confirmed the colocalization of TAGE and β-tubulin within the GCL and NFL (Figure 2C,F,I).

### 2.3. Pyridoxamine (PM) Dose-Dependently Inhibited TAGE Formation and TAGE–β-Tubulin Aggregation in the Retina after GA Treatment

**Figure 3:**
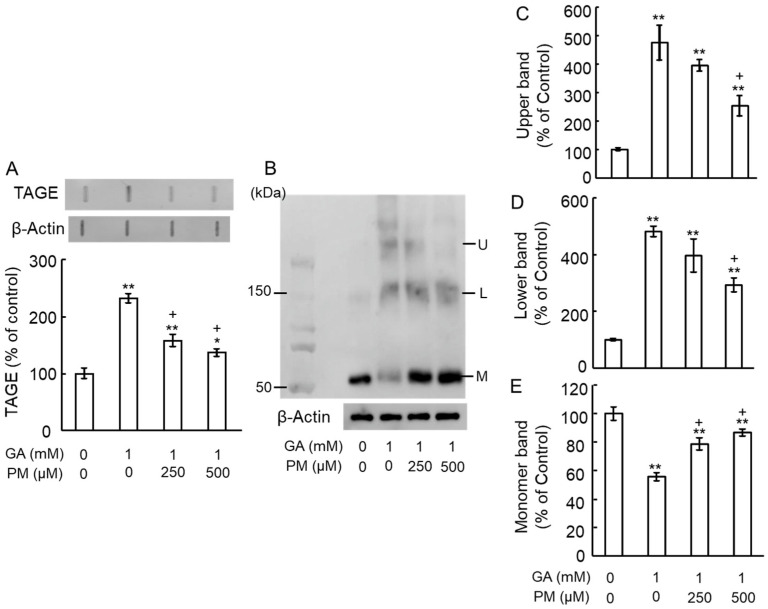
PM inhibited GA-induced TAGE formation and β-tubulin aggregation in the retina. (**A**) TAGE levels were measured using slot blot analysis with an anti-TAGE antibody at 3 days of treatment. The histogram shows the intensity of the TAGE bands in the slot blot. ** *p* < 0.01, * *p* < 0.05 vs. vehicle control. + *p* < 0.01 vs. GA alone (n = 3). (**B**) β-Tubulin levels detected using an anti-β-tubulin antibody (3-day treated retinal samples). U: Upper band, L: Lower band, M: Monomer band. (**C**–**E**) The intensity of the upper (**C**), lower (**D**), and monomer (**E**) β-tubulin bands upon GA treatment. ** *p* < 0.05 vs. vehicle control, + *p* < 0.01 vs. GA alone (n = 3).

We investigated the potential of PM, an AGE inhibitor, to counteract GA-induced TAGE formation and β-tubulin aggregation. Notably, PM at final concentrations of 250–500 μM alone did not affect TAGE levels or β-tubulin aggregation compared to the vehicle control. Slot blot analyses revealed a significant increase in TAGE formation upon GA treatment on day 3. This effect was dose-dependently attenuated by co-treatment with PM (Figure 3A). Similarly, Western blot analysis (Figure 3B) showed that GA decreased the levels of the 55 kDa monomeric β-tubulin band (Figure 3B,E) while increasing the intensity of the lower (Figure 3B,D) and upper (Figure 3B,C) bands indicative of aggregation. PM treatment significantly attenuated these GA-induced changes in a dose-dependent manner (Figure 3B–E).

### 2.4. PM Dose-Dependently Inhibited TAGE Formation in GCL and NFL after GA Treatment

**Figure 4:**
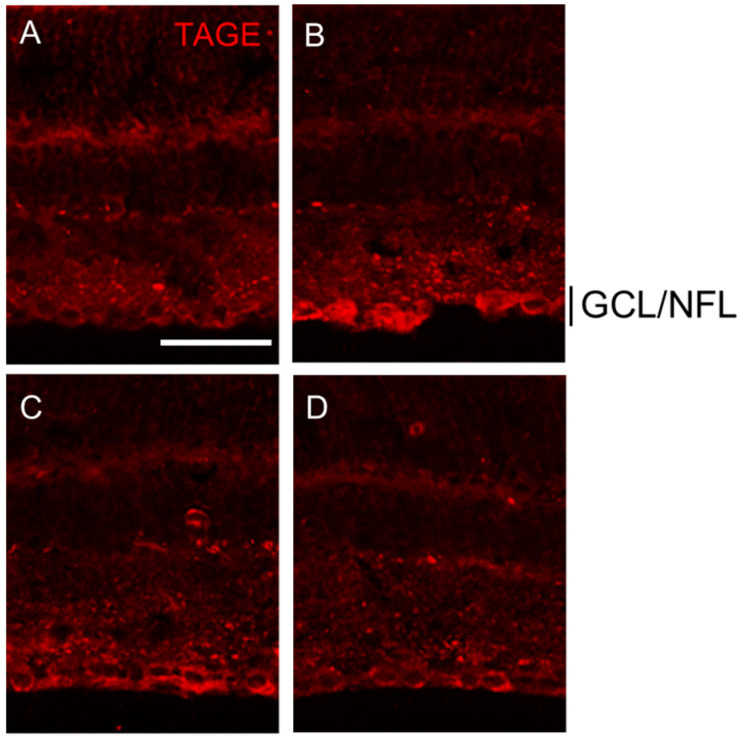
PM dose-dependently suppressed TAGE formation in GCL and NFL. (**A**–**D**) PM dose-dependently suppressed TAGE formation in GCL and NFL in retina. (**A**) 0 day, (**B**) GA, (**C**) GA plus 250 μM PM, (**D**) GA plus 500 μM PM. Scale = 100 μm.

PM showed dose-dependent suppression of TAGE levels in GCL and NFL. Intraocular injection of 1 mM GA on day 3 dramatically increased the TAGE levels in both the GCL and NFL compared to the vehicle control (Figure 4A vs. Figure 4B). Notably, PM treatment at concentrations of 250 μM (Figure 4C) and 500 μM (Figure 4D) strongly attenuated the TAGE levels in the GCL and NFL in a dose-dependent manner on day 3.

### 2.5. Zymosan Did Not Affect TAGE Formation and TAGE–β-Tubulin Aggregation by GA

**Figure 5:**
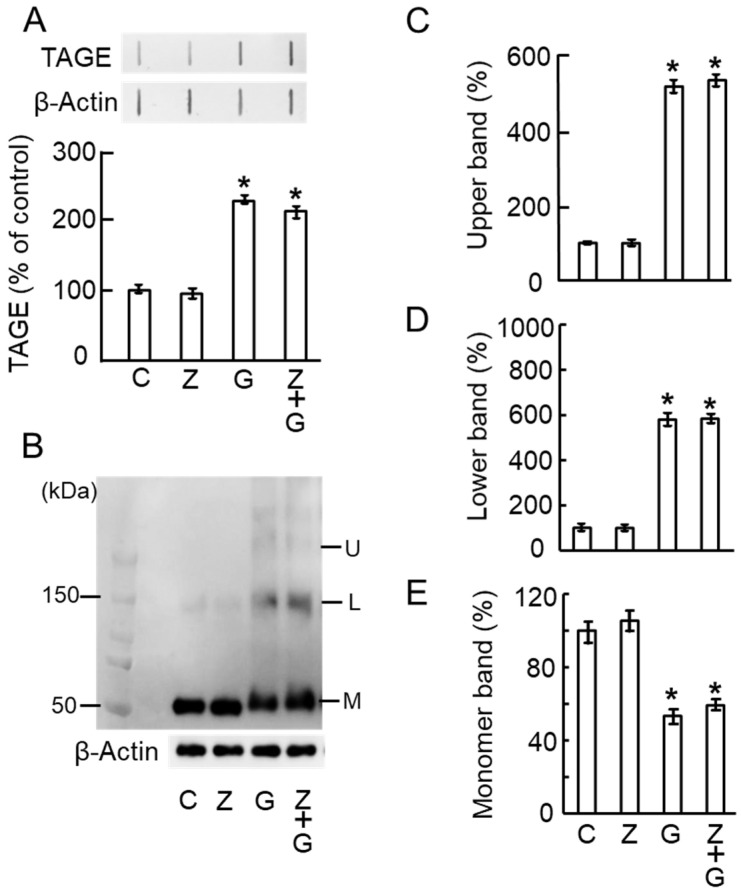
Zymosan did not affect TAGE formation and TAGE–β-tubulin aggregation by GA in the retina after optic nerve injury. (**A**) TAGE levels were measured using slot blot analysis with an anti-TAGE antibody. The histogram shows the intensity of the TAGE bands in the slot blot. * *p* < 0.01 vs. vehicle control (n = 3). (**B**–**E**) Levels of β-tubulin aggregation detected using an anti-β-tubulin antibody. (**B**) Western blot obtained using the anti-β-tubulin antibody. U: Upper band, L: Lower band, M: Monomer band. (**C**) The intensity of the upper β-tubulin band upon GA treatment. (**D**) The intensity of the lower β-tubulin band upon GA treatment. (**E**) The intensity of the monomer β-tubulin band upon GA treatment. * *p* < 0.01 vs. vehicle control (n = 3). C: vehicle control, Z: zymosan (12.5 μg/mL), G: GA (1 mM), Z+G: zymosan plus GA.

To confirm that zymosan, a potent activator of monocytes and promoter of axonal elongation [14,15], does not affect TAGE formation and β-tubulin aggregation by GA treatment, we administered GA and/or zymosan intravitreally and collected retinal samples 7 days after optic nerve injury. Zymosan alone did not affect the TAGE formation of the vehicle control. It also did not affect GA-induced TAGE formation (Figure 5A). On day 7 after optic nerve injury, vehicle treatment did not induce β-tubulin aggregation, and zymosan did not change the levels of the monomer, lower, and upper bands in the vehicle control treatment (Figure 5B–E). Moreover, zymosan did not change the levels of GA-induced β-tubulin aggregation, as evident from the levels of the monomer, lower, and upper bands (Figure 5B–E).

### 2.6. TAGE Inhibited Zymosan-Induced Axonal Elongation after Injury

**Figure 6:**
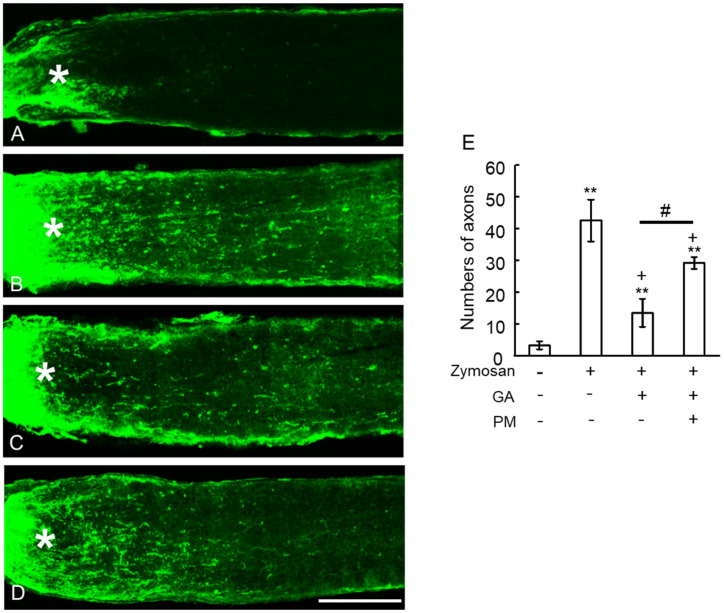
Axonal elongation induced by GA was dependent on TAGE. (**A**–**D**) Longitudinal sections of the adult mouse optic nerve showing GAP-43-positive axons extending over the injury site (asterisks) after 10 days of optic nerve injury. (**A**) Vehicle control, (**B**) Zymosan, (**C**) Zymosan plus GA, (**D**) Zymosan plus GA plus PM, (**E**) Quantification of axonal elongation at a point 250 μm distant from the injury site. ** *p* < 0.05 vs. vehicle control. + *p* < 0.05 vs. zymosan alone. # *p* < 0.01 vs. zymosan plus GA (n = 8, 6 mice per each group). Scale = 100 μm.

We further investigated the in vivo effects of GA on zymosan-induced axonal elongation following nerve injury (Figure 6A–E). Intraocular zymosan injection triggered axonal elongation as revealed by GAP43 staining, compared to the vehicle control (Figure 6B,E vs. Figure 6A,E). PM alone did not affect axonal elongation in the vehicle group. Interestingly, GA treatment significantly inhibited zymosan-induced axonal elongation compared to the vehicle control (Figure 6C,E). Furthermore, PM co-treatment significantly reversed the inhibitory effects of GA on axonal elongation (Figure 6D,E). These findings suggest a protective role for PM against GA-mediated suppression of axonal outgrowth.

### 2.7. GA Increased the Levels of Total Tau and Phosphorylation Tau in an AGE-Dependent Manner

**Figure 7:**
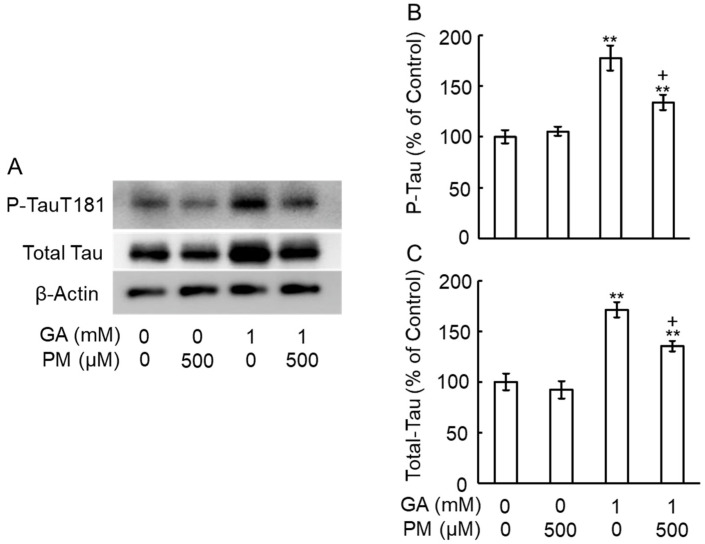
PM decreased the levels of phosphorylated tau induced by GA. (**A**) Western blot images showing the levels of total and phosphorylated tau (P-Tau). (**B**) Graphical representation of the intensity of Total-tau and (**C**) P-tau bands in the Western blot images shown in (**A**). ** *p* < 0.01 vs. vehicle control, + *p* < 0.01 vs. GA alone (n = 3).

Previous studies have shown increased intracellular tau phosphorylation in the cortex of patients with AD [16], and we previously reported similar findings in GA-treated SH-SY5Y cells [10]. To investigate whether PM could modulate GA-induced tau phosphorylation, retinas were pre-treated with PM for 30 min, followed by GA treatment for 3 days. Western blot analysis revealed significant increases in the levels of both total (Figure 7A,C) and phosphorylated (Figure 7A,B) tau upon GA treatment. PM co-treatment significantly attenuated the GA-induced increases in tau phosphorylation (Figure 7A–C), suggesting a protective role for PM against abnormal tau modifications.

## 3. Discussion

The alterations observed in the retina of patients with AD are linked to neuronal degeneration and loss, optic nerve degeneration, accumulation of amyloid-beta (Aβ) in the optic disk, and visual functional impairment [7,17,18]. Similar mechanisms of neurodegeneration observed in the brain have been reported in retinal neurons, including RGCs and the optic nerve, in the eyes of patients with AD [19]. In contrast, normal visual function relies on the proper functioning of retinal neurons; thus, the loss of neuronal function must underlie vision impairment in diabetes [20]. Recent studies suggest that retinal degeneration in diabetes may result not only from vasculopathy but also from neuropathy, encompassing both neuronal and axonal loss [21]. AGEs play a pivotal role in the progression of diabetic retinopathy, contributing to dysfunction and various losses of retinal neurons [22]. Moreover, the extent of AGE immunolabeling was higher in older donor eyes than that in younger ones [23]. Elevated levels of AGEs in RGCs and the optic nerve head signify axonal degeneration and visual loss, even in eyes affected by glaucoma [23].

Bikbova et al. reported that various AGEs, including Glu-AGEs and GA-AGEs, can induce apoptosis in retinal neurons and reduce the number of regenerating neurites in a retinal explant culture system [24]. Accumulating evidence suggests that cell death in retinal neurons and neurite abnormalities are linked to the early development of diabetic retinopathy [25]. However, the underlying mechanism remains elusive. This study aimed to explore the association between AD-related and AGE-dependent axonal degeneration in the optic nerve in DM. While most research on AD has centered on Glu-AGEs, we previously reported the critical involvement of α-hydroxyaldehydes, such as GA, a metabolic intermediate of Glu and fructose, and glycolaldehyde, including glyoxal, methylglyoxal, and 3-deoxyglucosone, in protein glycation. GA-AGEs exhibit higher cytotoxicity than other sugar-dependent AGEs, including Glu-AGEs [26]. Hence, GA-AGEs are also referred to as TAGE. TAGE have been detected in the axons and cytosol of neuronal cells in the hippocampus and parahippocampal gyrus of the brain tissues of patients with AD but not in senile plaques (SP) or glial cells [27]. In contrast, Glu-AGEs were found in SP amyloid cores and astrocytes. Additionally, TAGE were exclusively detected intracellularly, while Glu-AGEs were found both intra- and extracellularly. Based on this evidence, TAGE may represent a promising candidate for treating neurodegeneration in patients with AD.

In our previous study, GA was found to reduce Aβ42 levels in culture media [10]. Additionally, GA elevated the intracellular levels of total tau and phosphorylated tau in the human neuroblastoma cell line SH-SY5Y [10]. Through proteomics analysis using matrix-assisted laser desorption ionization-time of flight mass spectrometry, we identified β-tubulin as a target protein of TAGE. Microtubules, composed of α- and β-tubulin heterodimers, play a crucial role in neurite outgrowth. In our SH-SY5Y cell model, GA facilitated the formation of TAGE–β-tubulin and abnormal β-tubulin aggregation, resulting in the inhibition of axonal elongation [13]. Interestingly, β-tubulin can also undergo glycation by Glu in a DM experimental model [28]. However, under our experimental conditions, Glu did not induce abnormal β-tubulin aggregation or inhibit neurite outgrowth [13]. GA is derived from two distinct pathways, the glycolytic and fructose metabolic pathways [29]. In hyperglycemic conditions, increased intracellular Glu stimulates the polyol pathway to generate fructose in insulin-independent nerve tissue [30]. Fructose is further metabolized to dihydroxyacetone phosphate and GA by aldolase [31]. Consequently, GA production is promoted. Aldolase proteins are expressed in RGCs and other retinal cell layers [32]. However, a high-fructose diet has been shown to induce retinopathy by suppressing synaptic plasticity [33]. Elevated levels of the fructose transporter have been detected in diabetic retinopathy [34]. These findings suggest that both GA and TAGE are produced in the retinas of patients with diabetes.

Previous studies have demonstrated that PM, a natural form of vitamin B_6_, acts as an inhibitor of AGEs [35]. PM suppresses the formation of TAGE–β-tubulin and alleviates the GA-induced inhibition of axonal elongation. Vitamin B_6_ encompasses three subtypes: PM, pyridoxal, and pyridoxine. PM can trap aldehyde groups via its amino group, thereby preventing the formation of AGEs under physiological conditions. PM can effectively suppress AGE formation in various proteins both in vitro and in vivo, thereby preventing the development of diabetic complications such as hemoglobin glycation and lipoxidation reactions [36,37]. These compounds, including PM, serve as potent AGE inhibitors, attenuating diabetes-related nephropathy, neuropathy, and retinopathy [38]. Moreover, a deficiency in vitamin B_6_ is associated with abnormal nerve growth, contributing to conditions such as schizophrenia, depression, and central neuropathy [39]. Additionally, PM inhibits the early development of retinopathy in experimental diabetic models [40].

In this study, intraocular injection of GA increased the TAGE levels in the GCL and NFL. Furthermore, GA induced β-tubulin aggregation and inhibited zymosan-induced axonal elongation even in vivo. Benowitz et al. reported that zymosan induces macrophage invasion and appears to release trophic factors such as oncomodulin, stromal cell-derived factor 1, and CCL5 chemokine that can promote axonal elongation [41,42,43].

Tau proteins, which are integral components of paired helical filaments, exhibit distinct characteristics such as high aggregation propensity and hyperphosphorylation [44,45]. In neurodegenerative conditions such as AD, tau dissociates from microtubules within neurofibrillary tangles and aggregates in the cytosol, facilitating self-aggregation and phosphorylation [45]. Consequently, abnormal β-tubulin aggregation may increase the detachment of tau proteins from microtubules. While the precise mechanism remains unknown, the elevation in total tau levels is believed to stem from axonal loss [46]. Recent studies have highlighted GA’s ability to induce AD-like alterations in vitro [47,48]. Piccirillo et al. documented GA-induced phosphorylation of tau at T212 and T214, while another study illustrated the impact of GA on tau phosphorylation at S199 and S396, alongside reduced axonal outgrowth [49,50]. Consequently, tau proteins become hyperphosphorylated at various sites, aggregating into neurofibrillary tangles within AD patient brains. Notably, tau phosphorylation at T181, T217, and T231 in CSF is a promising biomarker for AD diagnosis [51]. T217 and T231 are indicative of postsynaptic pathology, while T181 marks axonal abnormalities [52]. In our recent study, TAGE–β-tubulin accelerated abnormal aggregation and hindered neurite outgrowth, accompanied by T181 phosphorylation [10,13]. Similar outcomes were observed in the retinal optic nerve model. Further investigations are warranted since the detailed mechanisms underlying GA-induced β-tubulin aggregation and tau phosphorylation remain elusive.

While our study provides valuable insights into the pathogenic mechanisms underlying visual dysfunction in DM and AD, several limitations warrant consideration. First, the use of animal models may not fully capture the complexity of human disease progression, potentially limiting the translational relevance of our findings. Additionally, our study focused on a single time point analysis, precluding a comprehensive assessment of the long-term effects of GA-induced β-tubulin aggregation and neurite outgrowth inhibition. Furthermore, while we elucidated the involvement of AGEs in mediating neurodegeneration, the specific molecular mechanisms remain incompletely understood. Addressing these limitations in future research endeavors could provide a more comprehensive understanding of visual dysfunction in these debilitating conditions.

## 4. Materials and Methods

### 4.1. Ethics Statement

All animal care and handling procedures were approved by the Animal Care and Use Committee of Suzuka University of Medical Science (No. 78). All surgeries were performed under sodium pentobarbital anesthesia, and efforts were made to minimize suffering.

### 4.2. Chemicals

Zymosan, an axonal elongation inducer, was purchased from Sigma-Aldrich (Tokyo, Japan). The AGE inhibitor pyridoxamine was purchased from Sigma-Aldrich (Tokyo, Japan).

### 4.3. Preparation of Anti-TAGE Antibody

Immunoaffinity-purified anti-TAGE antibodies were prepared according to previously established methods [53]. Despite their specificity, these antibodies did not recognize well-characterized AGE structures, including triosidines, GA-derived pyridinium compounds, GA-derived pyrrolopyridinium lysine dimers, methylglyoxal-derived hydroimidazolone-1, and argpyrimidine. Moreover, the antibodies failed to detect AGEs with unknown structures, such as Glu-AGEs and fructose-derived AGEs. However, they exhibited specific recognition of unique dimeric/trimeric TAGE structures [54].

### 4.4. Animals and Surgery

Male C57BL6 mice aged 8–9 weeks were utilized for this study. The mice were administered sodium pentobarbital intraperitoneally at a dosage of 30–40 mg/kg and xylazine at 5 mg/kg. Intraocular injections of various reagents were administered into the sclera and retina using a 30 G needle positioned 1–2 mm superior to the optic nerve head to prevent lens injury [55]. The injection volume was 3 μL after pre-suction of the same volume of vitreous fluid. Optic nerve injury was induced 2 mm behind the eye using angle jeweler forceps (Dummont #5) for a duration of 10 s [55]. The mice were housed in clear plastic cages and maintained under a 12 h light/dark cycle at 23 °C. PM and/or zymosan were administered intraocularly 30 min prior to GA injection.

### 4.5. Immunohistochemistry

Tissue fixation and cryosectioning were performed as previously described [56]. Briefly, the eyes were enucleated and fixed in 4% paraformaldehyde in 0.1 M phosphate buffer (pH 7.4) and cryoprotected in 20% sucrose. The eyes and optic nerves were then embedded in an optimal cutting temperature compound (TissueTek; Miles, Eikhart, IN, USA) and cryosectioned at a thickness of 12 μm (retina) and 16 μm (optic nerve). The frozen sections were mounted onto silane-coated glass slides. After washing and blocking with Blocking One (NakalaiTesque, Kyoto, Japan), the sections were incubated overnight with various antibodies, such as those against β-tubulin (1:500, Cell Signaling Technology, Tokyo, Japan), TAGE (1:500), and GAP43 (1:250) at 4 °C. The sections were then incubated with secondary fluorescent anti-IgG (Thermo Fisher Scientific, Tokyo, Japan) at 23 °C (1:1000, Alexa Fluor 488 anti-rabbit IgG for β-tubulin, 1:1000, Alexa Fluor 594 anti-rabbit IgG for TAGE, and 1:500, Alexa Fluor 488 anti-sheep IgG for GAP43).

### 4.6. Slot Blot Analysis

To assess TAGE levels, we performed a slot blot analysis. Retinal samples were isolated and homogenized on each day of GA injection. Equal amounts of protein (30 μg) were applied to nitrocellulose membranes by vacuum filtration. The membranes were blocked with Blocking One for 1 h at 23 °C and then incubated with an anti-TAGE antibody. The positive bands were detected using a BCIP/NBT kit (Sera Care, Milford, MA, USA) and analyzed densitometrically as previously described [57]. All experiments were repeated at least thrice.

### 4.7. Western Blot Analysis

Retinal tissue proteins were extracted, and sample aliquots (30 μg) were subjected to polyacrylamide electrophoresis using a 5–20% gradient gel, as previously described [58]. The separated proteins were transferred to a nitrocellulose membrane and incubated with Blocking One, and then the primary antibodies (all at 1:500): anti-β-tubulin and anti-total tau or anti-phosphorylated tau (Cell Signaling Technology, Tokyo, Japan). The secondary antibody was horseradish peroxidase-labeled anti-IgG (1:1000; Proteintech, Tokyo, Japan). The chemiluminescent HRP substrate (Millipore, Burlington, MA, USA) was used to detect the protein bands. The bands were densitometrically analyzed using Scion Image Software 4.0.3.2 (Scion Corp., Frederick, MD, USA). All experiments were performed at least in triplicate.

### 4.8. Quantitation of Axonal Elongation of Optic Nerve In Vivo

The mice were sacrificed 10 days after optic nerve injury and perfused with 4% paraformaldehyde. Optic nerves were impregnated with 5% and then 20% sucrose, embedded in an optimal cutting temperature compound (Sakura Fine Technical, Tokyo, Japan), and cut into longitudinal sections of 10 μm thickness. Axonal elongation was visualized by staining with a sheep anti-GAP43 antibody (1:250, the antibody was kindly provided by Dr. Benowitz, Children’s Hospital, Harvard Medical School, Boston, MA, USA), followed by a fluorescently-labeled secondary antibody, and captured by fluorescence microscopy (BZ-9000, Keyence, Osaka, Japan). Axons were counted manually in at least eight sections per condition (six mice per treatment) at prespecified distances (250 μm) from the injury site. The numbers of axonal elongation were counted as described by Yin et al. [59].

### 4.9. Statistics

All results were presented as mean ± standard error of the mean (SEM) for 3–5 experiments. Differences between groups were analyzed using one-way ANOVA followed by Dunnett’s multiple comparison test, performed using PASW Software version 28 (SPSS Inc., Chicago, IL, USA). Statistical significance was considered at *p* < 0.05.

## 5. Conclusions

The present study demonstrates that GA induces the aggregation of β-tubulin through an AGE-dependent mechanism, resulting in the inhibition of neurite outgrowth even in vivo. Therefore, TAGE–β-tubulin emerges as a promising target for understanding the pathogenic mechanisms contributing to visual dysfunction in both DM and AD. Our findings support the notion that therapies aimed at reducing AGE formation, such as supplementation with preventive medications such as AGE inhibitors, may suppress neurodegeneration in the visual systems affected by both DM and AD.

## Data Availability

The data supporting the findings of this study are available upon request from the corresponding author.
